# Antimicrobial Peptide Mastoparan-AF Kills Multi-Antibiotic Resistant *Escherichia coli* O157:H7 via Multiple Membrane Disruption Patterns and Likely by Adopting 3–11 Amphipathic Helices to Favor Membrane Interaction

**DOI:** 10.3390/membranes13020251

**Published:** 2023-02-20

**Authors:** Chun-Hsien Lin, Ching-Lin Shyu, Zong-Yen Wu, Chao-Min Wang, Shiow-Her Chiou, Jiann-Yeu Chen, Shu-Ying Tseng, Ting-Er Lin, Yi-Po Yuan, Shu-Peng Ho, Kwong-Chung Tung, Frank Chiahung Mao, Han-Jung Lee, Wu-Chun Tu

**Affiliations:** 1 Department of Entomology, National Chung Hsing University, Taichung 40227, Taiwan; 2 Graduate Institute of Microbiology and Public Health, National Chung Hsing University, Taichung 40227, Taiwan; 3 Department of Veterinary Medicine, National Chung Hsing University, Taichung 40227, Taiwan; 4 Department of Veterinary Medicine, National Chiayi University, Chiayi 60054, Taiwan; 5 i-Center for Advanced Science and Technology, National Chung Hsing University, Taichung 40227, Taiwan; 6 Veterinary Medical Teaching Hospital, National Chung Hsing University, Taichung 40227, Taiwan; 7 Department of Natural Resources and Environmental Studies, National Dong Hwa University, Hualien 974301, Taiwan; 8 National Mosquito-Borne Diseases Control Research Center, National Health Research Institutes, Kaohsiung 801301, Taiwan; 9 School of Life Sciences and Technology, Institut Teknologi Bandung, Bandung 40132, West Java, Indonesia

**Keywords:** antibiotic resistance, antimicrobial peptide, *Escherichia coli* O157:H7, mastoparan-AF, membrane disruption pattern, membrane permeabilization, *Vespa affinis*

## Abstract

We investigated the antimicrobial activity and membrane disruption modes of the antimicrobial peptide mastoparan-AF against hemolytic *Escherichia coli* O157:H7. Based on the physicochemical properties, mastoparan-AF may potentially adopt a 3–11 amphipathic helix-type structure, with five to seven nonpolar or hydrophobic amino acid residues forming the hydrophobic face. *E. coli* O157:H7 and two diarrheagenic *E. coli* veterinary clinical isolates, which are highly resistant to multiple antibiotics, are sensitive to mastoparan-AF, with minimum inhibitory and bactericidal concentrations (MIC and MBC) ranging from 16 to 32 μg mL^−1^ for *E. coli* O157:H7 and four to eight μg mL^−1^ for the latter two isolates. Mastoparan-AF treatment, which correlates proportionally with membrane permeabilization of the bacteria, may lead to abnormal dents, large perforations or full opening at apical ends (hollow tubes), vesicle budding, and membrane corrugation and invagination forming irregular pits or pores on *E. coli* O157:H7 surface. In addition, mRNAs of prepromastoparan-AF and prepromastoparan-B share a 5′-poly(A) leader sequence at the 5′-UTR known for the advantage in cap-independent translation. This is the first report about the 3–11 amphipathic helix structure of mastoparans to facilitate membrane interaction. Mastoparan-AF could potentially be employed to combat multiple antibiotic-resistant hemolytic *E. coli* O157:H7 and other pathogenic *E. coli*.

## 1. Introduction

The emergence of multiple antibiotic-resistant bacteria, notably, pan-resistant Gram-negative pathogens, which are equipped with an outer membrane barrier of low permeability to antibiotics, has become an important challenge in recent decades following the overuse of antibiotics in humans and animals [1]. In particular, the foodborne enteric pathogen *Escherichia coli* O157:H7 has caused severe or deadly illness cases worldwide [2,3]. Among *E. coli* O157 isolates, serotype O157:H7 is the most common enteric pathogen isolated from patients with bloody diarrhea and it is also frequently found in non-bloody diarrhea samples [2,3]. Many of its clinical isolates from humans and animals as well as isolates from contaminated food have been found to develop resistance to several antibiotics [4]. In recent years, both O157 and non-O157 drug-resistant diarrheagenic *E. coli* strains are receiving comparably epidemiological interests and are of zoonotic concern [2,5]. Drug-resistant *E. coli* strains that are predominant in diarrhetic dogs, especially, may have a chance to spread to humans due to close contact and should not be overlooked [5]. Therefore, it is imperative to seek an alternative treatment to control drug-resistant *E. coli* O157:H7 and other *E. coli* diarrheagenic pathogens. Mastoparans, as a candidate group of cationic antimicrobial peptides [6,7], should be considered. Following the first isolation of mastoparan, the most abundant peptide in the hornet or wasp venom [7,8], from *Vespula lewisii* [9], many homologs of mastoparan were isolated from various hornets and solitary wasps [10,11,12,13,14,15]. Mastoparan homologs are cationic tetradecapeptides with membrane permeabilizing activity and antimicrobial activity on various bacteria [6,15,16], mast cell degranulation activity [9,10,11], and hemolytic activity [6,13]. Our previous study has shown that mastoparan from hornet venom of *Vespa affinis*, designated as mastoparan-AF, presented with superior antibacterial activity (with lower MIC or MBC than five other mastoparan homologs) against several pathogens, including *Staphylococcus aureus*, *Klebsiella pneumonia, Pseudomonas aeruginosa, Salmonella typhimurium*, and *Vibrio parahamelytics* [6]. Nonetheless, the actual effect and any mechanistic actions of mastoparan-AF on *E. coli* O157:H7 remain to be deciphered.

Circular dichroism spectra have revealed that mastoparans adopt a disordered conformation in water but form helical structures in synthetic lipids (such as sodium dodecyl sulfate, SDS) and artificial membranes [6,17]. Many cationic antimicrobial peptides can fold into amphipathic (or amphiphilic) structures, with both hydrophilic (positively charged) and hydrophobic domains, when in contact with membranes [18]. In fact, amphipathic helices represent one important structural feature of cationic antimicrobial peptides among several other amphipathic structures [18]. Previous studies indicate that the formation of helical structures is important for the biological activities of mastoparans [17,19]. It has been generally assumed that mastoparans turn into amphipathic α-helix in membrane environments [17]. However, several studies on amphipathic helices of proteins unveil that, apart from α-helix, which comprises 3.6 amino acid residues (aa) per turn (i + 4), other helices may be present in various membrane proteins [20]. Among them, 3–10 helix (3.3 aa per turn, i + 3) [21]), 3–11 helix (3.67 aa per turn) [20], and π-helix (4.4 aa per turn, i + 5) [22] have smaller, slightly wider, and wider radius than α-helix, respectively, and each may have different impacts on membrane interaction. Therefore, we should compare and explore whether α-helix or any other helices may likely account for the membrane interacting ability of mastoparans, which is essential for many of their biological activities (e.g., membrane permeabilization).

Simulation studies, using computer models to mimic the molecular actions of membrane disruptive antimicrobial peptides on artificial lipid bilayer membranes or vesicles, have been widely used to explore their membrane disruption patterns [23,24]. Membrane disruption models, in particular, barrel-stave, toroidal pore, and carpet models have been proposed as the major molecular mechanisms to account for the membrane disruptive actions of antimicrobial peptides [23,24,25]. In addition to simulation methods, many studies have employed atomic force microscopy (AFM), a powerful tool for imaging membrane changes at nanometer-level resolution, to investigate the impacts and action modes of antimicrobial peptides on model lipid bilayers or membranes [26]. Antimicrobial peptides selectively damage anionic model membranes via variable and distinguishable action modes, and each mode conforms with one of the membrane disruption models. Except for very few multi-structural peptides exhibiting multi-modal actions, most single-structure antimicrobial peptides usually present with single membrane disruption modes toward model membranes, which can be influenced by peptide concentration, lipid compositions, and fluidity of the interacting membranes [26]. However, more comprehensive AFM studies exploring the action modes of antimicrobial peptides on actual pathogens, which have much more diverse and complicated lipid compositions than model membranes, are limited [26].

In this study, we explored the impacts and mechanistic actions of mastoparan-AF on the drug-resistant foodborne enteric pathogen *E. coli* O157:H7, and two diarrheagenic *E. coli* isolates from dogs, which, as mentioned above, are potentially zoonotic and urgently in need of alternative treatment. The full-length cDNA encoding the complete coding sequence (CDS) of prepromastoparan-AF, the precursor polypeptide of mastoparan-AF, was cloned. In addition, mastoparan-AF was examined for its antibacterial effect against hemolytic *E. coli* O157:H7, along with *S. aureus*, and two clinical isolates of *E. coli*. The sensitivities/or resistances of these bacteria toward multiple classes of antibiotics were compared in parallel. Membrane permeabilization on hemolytic *E. coli* O157:H7 and hemolytic activities on human, chicken, and sheep erythrocytes (red blood cells, RBCs) were determined. Furthermore, we observed the surface disruption patterns of mastoparan-AF on hemolytic *E. coli* O157:H7 by scanning electron microscopy (SEM) and AFM. By using Heliquest online software, we analyzed and compared the physicochemical properties of mastoparan-AF under different helical structures.

## 2. Materials and Methods

### 2.1. Biological Materials

Worker hornets of *V. affinis* captured from the fields of central Taiwan were paralyzed at 4 °C. Their venom glands were dissected from the abdomens in sterile DEPC-treated phosphate buffer and transferred into the RNA extraction solution, Tri Reagent (Molecular Research Center, Cincinnati, OH, USA), at −70 °C until used.

### 2.2. Cloning of a Full-Length cDNA Fragment Encoding the Precursor Polypeptide of Mastoparan-AF

The total RNA of the venom gland of *V. affinis* was isolated using Tri Reagent (Molecular Research Center, Cincinnati, OH, USA). The full-length cDNA encoding the precursor polypeptide of mastoparan-AF, prepromastoparan-AF, was obtained by using a Smart^TM^ RACE cDNA amplification kit (BD Biosciences Clontech, Palo Alto, CA, USA). Two specific primers, MP-AF-F (5′-GCTATTGCAGCATTGGCTAAGAAA-3′) and MP-AF-R (5′-CAATGCTGCAATAGCCTTCAG-3′) were synthesized, respectively, according to the amino acid sequence of mastoparan-AF and other mastoparans [6,14,15,27]. The amplified reverse transcription-polymerase chain reaction (RT-PCR) products were cloned into the pGEM-T Easy Vector (Promega, Madison, WI, USA) and sequenced. The cDNA sequence is deposited in *Gen*Bank (accession no. HQ156227).

### 2.3. Peptide Synthesis

Mastoparan-AF was synthesized and amidated at its C-terminus, INLKAIAALAKKLF-NH_2_, with a purity of 95% by Genomics BioSci & Tech (Taipei, Taiwan, ROC). The identity of mastoparan-AF was verified by electrospray ionization mass spectrometry (ESI-MS) (Q-Exactive Plus Mass Spectrometer, Thermo Fisher Scientific, Waltham, MA, USA). The synthetic mastoparan-AF was stored at −70 °C until use.

### 2.4. Membrane Permeabilization Assay

Membrane permeabilization assay was performed by measuring the β-galactosidase activity of bacteria as described previously [6]. Briefly, hemolytic *E. coli* O157:H7 grown at 37 °C for 16 h in LB medium containing 1% lactose was washed and the bacterial cell suspension (10^7^ CFU mL^−1^) was incubated in various concentrations of mastoparan-AF (in 54 μL of 130 mM NaCl and 10 mM sodium phosphate buffer at pH 7.0) in a 96-well round bottom microtiter plate at 37 °C for 30 min. Subsequently, ο-nitrophenyl-β-D-galactoside (ONPG) (final 1.5 mM in a total volume of 60 μL) was added to each well and incubated at 37 °C for 2.5 h. The reaction was terminated by adding 10 μL of 0.8 N NaOH. The rate of membrane permeabilization was based on the rate of ο-nitrophenol (ONP) production. The absorbance was measured at 405 nm (yellow). The 0 and 100% membrane permeabilization were determined in the presence of 10 mM sodium phosphate buffer (pH 7.0, containing 130 mM NaCl) and 1% Triton X-100, respectively. The membrane permeabilization (%) was calculated using the following equation: the membrane permeabilization (%) = [(*A*_mastoparan-AF_ − *A*_sodium phosphate_)/(*A*_1% Triton X-10_ − *A*_sodium phosphate_)] × 100%. Results were expressed as means ± SD of four replicates.

### 2.5. Hemolytic Activity Assay

Hemolytic activity assay [28] was performed with modification as described earlier [6]. Sheep blood (defibrinated) was commercially available (Taiwan Prepared Media, TPM, Taipei, Taiwan). Chicken blood was collected using BD Vacutainer^TM^ tubes (Becton Dickinson, Franklin Lakes, NJ, USA) containing the anticoagulant sodium heparin. The permission for the experiment was granted by the Institutional Animal Care and Use Committee of National Chung Hsing University (IACUC of NCHU), Taichung, Taiwan (IACUC Approval No. 101001, approved on 14 June 2012). Blood samples were centrifuged at 1500× *g* for 10 min. The erythrocytes (red blood cell, RBC) pellets were washed three times with phosphate-buffered saline (PBS, pH 7.4) and resuspended at 10% of the original concentration in the same buffer. One hundred μL of 10% RBC suspension was mixed gently with 100 μL of various concentrations of mastoparan-AF and incubated at 37 °C for 30 min. After centrifugation at 1500× *g* for 10 min, the supernatant was carefully transferred to the 96-well round bottom microtiter plate and measured at 570 nm. The 0 and 100% hemolysis were determined in the presence of PBS and 0.1% Triton X-100, respectively. The hemolysis (%) was determined using the following equation: Hemolysis (%) = [(*A*_mastoparan-AF_ − *A*_PBS_)/(*A*_0.1% Triton X-100_ − *A*_PBS_)] × 100%. Results were expressed as means ± SD of four replicates.

### 2.6. Antimicrobial (Antibacterial) Activity Assay of Mastoparan-AF

The bacterial strains tested were as follows, *Staphylococcus aureus* subsp. *aureus* (ATCC 33591), *E. coli* JM109 pAcUW21 (carrying ampicillin resistance gene, *Amp^R^*), hemolytic *E. coli* O157:H7 (ATCC 43894), and two hemolytic clinical isolates (232 and 237) of *E. coli* isolated from fecal samples of diarrhetic outpatient dogs (Veterinary Medical Teaching Hospital, National Chung Hsing University, Taichung, Taiwan, ROC). Each bacterial strain was grown in a liquid tryptic soy broth (TSB, Merck, Darmstadt, Germany) medium to the exponential phase, and the bacterial suspension was adjusted to 10^5^ to 10^6^ colony-forming units (CFU) mL^−1^.

The antimicrobial (antibacterial) activity assay [28,29] of mastoparan-AF was performed in three independent experiments in duplicate, as described previously [6]. One hundred μL of bacterial suspension described above was added and incubated with different concentrations (serial 2-fold dilutions) of mastoparan-AF in phenol red broth containing 1% glucose in each well (final 200 μL) of the 96-well round bottom plate at 37 °C for 24 h. Bacterial growth was analyzed based on both colorimetric observation and OD_590_ measurement. The minimal inhibitory concentration (MIC), defined as the lowest concentration of mastoparan-AF that completely inhibited bacterial growth, was recorded after 24 h incubation. MIC interpretive criteria were based on the antimicrobial susceptibility testing standards of the Clinical and Laboratory Standards Institute (CLSI). The minimum bactericidal concentration (MBC) was determined by subculturing mastoparan-AF-treated bacteria on tryptic soy agar (TSA) plates.

### 2.7. Antibiotic Susceptibility Assay

Bacterial strains were described as above (2.6). The antibiotic susceptibility or resistance assay was determined by the broth microdilution method following a standard procedure from the CLSI. Bacterial suspension was incubated with different concentrations (serial 2-fold dilutions) of antibiotics in Mueller Hinton broth (200 μL/well) in a 96-well round bottom plate at 37 °C for 18 h. Bacterial growth was analyzed based on OD_590_ measurement. The MIC is defined as the lowest concentration of an antibiotic that completely inhibited bacterial growth. The CLSI performance standards for Antimicrobial Susceptibility Testing (M100-S30) were used for interpreting the MIC values.

### 2.8. Scanning Electron Microscopy (SEM)

Hemolytic *E. coli* O157:H7 was grown to the exponential phase in liquid TSB medium. Bacteria were treated with mastoparan-AF at 2 × MIC (32 μg mL^−1^) for 1 h at room temperature and harvested by centrifugation. Pelleted bacteria were fixed with 0.5% glutaraldehyde and sequentially dehydrated in 70, 80, 90, 95, and 100% ethanol. After critical point drying, bacteria were coated with pure gold and examined by an Inspect^TM^ S50 scanning electron microscope (FEI Company, Hillsboro, OR, USA).

### 2.9. Atomic Force Microscopy (AFM)

Hemolytic *E. coli* O157:H7 was grown to the exponential phase in liquid TSB medium. Bacteria were treated with mastoparan-AF at 2 × MIC (32 μg mL^−1^) for 1 h and fixed directly (without centrifugation) with 2.5% glutaraldehyde at 4 °C overnight. The bacteria were sequentially dehydrated in 70%, 80%, 90%, and 100% ethanol. After drying, the topography of bacteria was measured by a commercial atomic force microscope (Dimension Icon) (Bruker, Billerica, MA, USA), and the tapping mode was engaged at room temperature under atmospheric environment. The atomic force microscope probe was adopted from NCSTR series (Nanoworld, Neuchatel, Switzerland) with a resonance frequency of 160 kHz and a spring constant of 7.4 N/m, respectively. For image quality, the scan rates of the tip were 0.3–0.6 Hz, with a resolution set of 512 by 256 pixels, and the feedback control parameters were optimized. The 3D topography of bacteria was analyzed using the NanoScope Analysis software (Version 7.4).

### 2.10. Sequence Alignment

Sequence alignment was performed using the Basic Local Alignment Search Tool (BLAST) (National Center for Biotechnology Information, United States National Library of Medicine) available on the internet (https://blast.ncbi.nlm.nih.gov/Blast.cgi), accessed on 17 April 2017.

### 2.11. Physicochemical Property Analysis

Physicochemical properties of mastoparans were analyzed by using an online software, Heliquest (https://heliquest.ipmc.cnrs.fr/), accessed on 4 July, 9 July and 26 July 2021.

## 3. Results

### 3.1. Cloning of the Full-Length cDNA Fragment Encoding Prepromastoparan-AF

**Figure 1:**
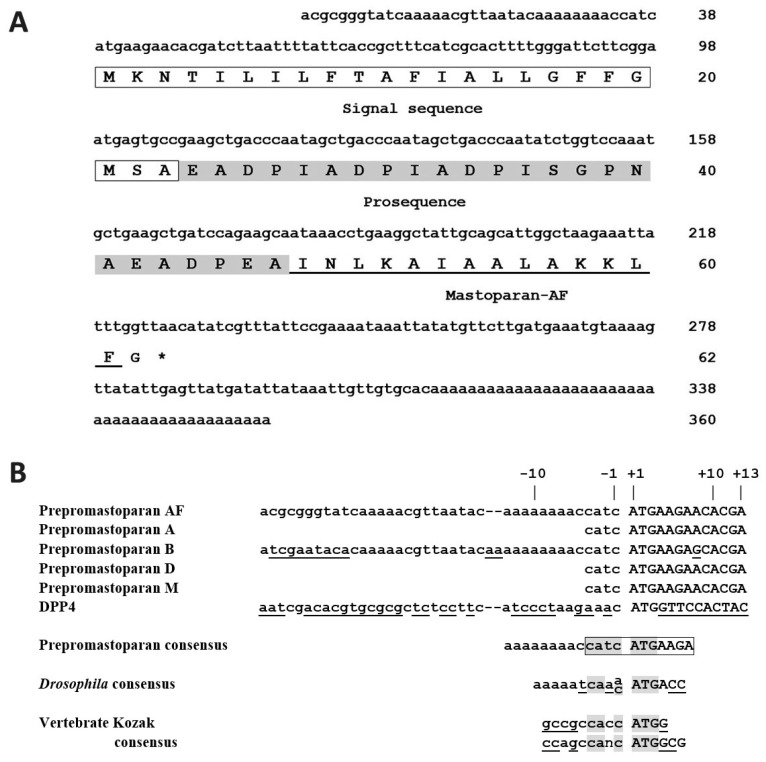
The cDNA sequence encoding the prepromastoparan-AF and alignment of the 5′ prepromastoparan mRNAs sequences. (**A**) The cDNA sequence (lowercase letters) encoding the prepromastoparan-AF. Number of the last nucleotide or amino acid residue in each line is labeled on the right. The amino acid sequence (1-letter abbreviations) of signal sequence, prosequence, and mature mastoparan-AF of prepromastoparan-AF is boxed, shaded, and underlined, respectively. Asterisk indicates the stop codon, TAA. (**B**) Alignment of the 5′ prepromastoparan mRNAs sequences (open reading frame in uppercase). The prepromastoparan-AF mRNA sequence flanking the translation initiation region was aligned against those of other homologs, including prepromastoparan-A from *Vespa analis* [6], prepromastoparan-B from *Vespa basalis* (DQ119291) [6,27], prepromastoparan-D from *Vespa ducalis* [6], and prepromastoparan-M from *Vespa mandarinia* [6]. DPP4 from *V. basalis* (DQ661743) [27] is listed in parallel. An 11-bp “CATCATGAAGA” sequence that is identical among all prepromastoparan homologs is boxed. In addition, prepromastoparan-AF and prepromastoparan-B mRNA sequences are identical from -13 to +7 (5′-AAAAAAAACCATCATGA AGA). A core consensus sequence (−4 to +3) that is conserved among prepromastoparan mRNAs, *Drosophila* consensus [30], vertebrate Kozak [31], and similar consensus [30] sequences are highlighted in gray. Nucleotides varied from prepromastoparan-AF are underlined.

Previously, we cloned a partial cDNA of the precursor polypeptide of mastoparan-AF, prepromastoparan-AF [6]. In this study, to investigate both 5′ untranslated and 3′ untranslated regions, the full-length cDNA of prepromastoparan-AF from *V. affinis* was obtained by employing the rapid amplification of cDNA ends (RACE) technique and the sequence was deposited in *Gen*Bank (accession no. HQ156227). As shown in Figure 1A, the 360 bp cDNA fragment, which fulfills the amino acid sequence of prepromastoparan-AF, encodes a signal sequence of 23 amino acid residues, an anionic prosequence of 24 amino acid residues, the mature mastoparan-AF of 14 amino acid residues, and an appendix glycine at C-terminus.

The nucleotide sequence of 5′ prepromastoparan-AF mRNA was compared with those of other prepromastoparan homologs, including prepromastoparan-A from *Vespa analis* [6], prepromastoparan-B from *Vespa basalis* [6,27], prepromastoparan-D from *Vespa ducalis* [6], and prepromastoparan-M from *Vespa mandarinia* [6]. The sequence alignment revealed an 11-bp conserved sequence “CATCATGAAGA” from −4 to +7 near the translation initiation codon among prepromastoparan mRNAs (Figure 1B). In fact, prepromastoparan-AF and prepromastoparan-B mRNA sequences are identical from −13 to +7 (5′-AAAAAAAACCATCATGAAGA). *Drosophila* [30] and vertebrate (including Kozak) [30,31] consensus sequences flanking the translation initiation region, along with the mRNA sequence of dipeptidyl peptidase IV (DPP4) from *V. basalis* (DQ661743) [27], were compared in parallel. Within −10 to +4, prepromastoparan-AF and prepromastoparan-B mRNAs share an identical sequence, which, being quite different from that of DPP4 (Figure 1B), is very similar to *Drosophila* consensus and similar in part (−4 to +3) with vertebrate consensus sequences [30,31].

### 3.2. Using a Synthetic Mastoparan-AF to Measure Its Membrane Permeabilization Activity

**Figure 2:**
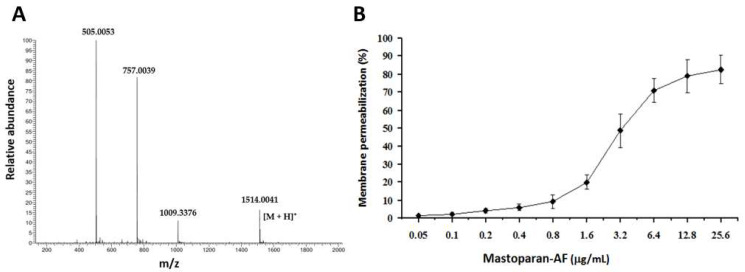
Mass spectrometry (MS) and membrane permeabilization effect of mastoparan-AF on hemolytic *E. coli* O157:H7. (**A**) Mastoparan-AF was synthesized and the identity was confirmed by MS. (**B**) Membrane permeabilization assay was performed by measuring the β-galactosidase activity of hemolytic *E. coli* O157:H7, using ο-nitrophenyl-β-D-galactoside (ONPG) as the substrate. The absorbance values measured at 405 nm in the presence of sodium phosphate and 1% Triton X-100 were determined as 0 and 100% membrane permeabilization, respectively. Results were expressed as means ± SD (*n* = 8).

Considering that it is relatively easy and cost-effective to grow bacteria, efforts were made to express mastoparan-AF in a prokaryotic system, but we did not observe any recombinant peptide expression. Therefore, we chose to use a synthetic mastoparan-AF to proceed with its activity assays in this study. Similar to other mastoparans, native mastoparan-AF is amidated in its C-terminus [6,7,12,15,27,32]. The C-terminal amidation is known to facilitate the helical structure formation and membrane interaction [7]. Mastoparan-AF was synthesized with C-terminal amidation and the identity was confirmed by ESI-MS (Figure 2A).

The membrane permeabilization effect of mastoparan-AF on hemolytic *E. coli* O157:H7 was measured by a membrane permeabilization assay [6]. If mastoparan-AF could cause membrane permeabilization of bacteria, ONPG (a colorless chromogenic substrate of β-galactosidase) would enter the cytoplasm and be hydrolyzed by cytosolic β-galactosidase to generate ONP (yellow). Figure 2 shows that mastoparan-AF caused membrane permeabilization on hemolytic *E. coli* O157:H7 in a dose-dependent manner. At 3.2 μg mL^−1^, mastoparan-AF led to substantial membrane permeabilization, with the production of ONP approaching 50% of the positive control (1% Triton X-100 treatment). As the concentration increased to 12.8 or 25.6 μg mL^−1^, mastoparan-AF caused around or beyond 80% of membrane permeabilization of the positive control (Figure 2B).

### 3.3. Limited Hemolytic Activity on RBCs

**Figure 3:**
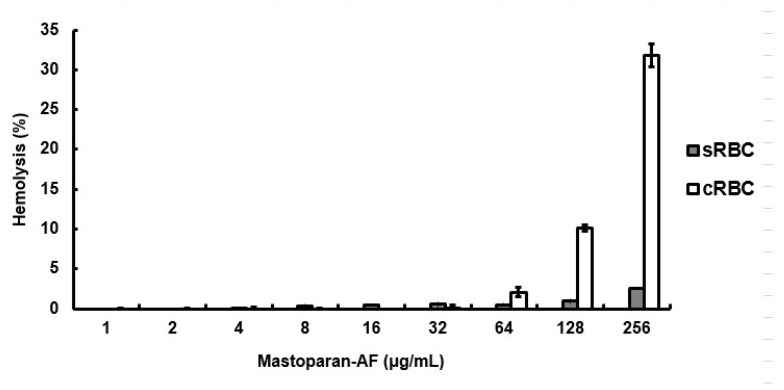
Hemolytic activity of mastoparan-AF on RBCs. The hemolytic activity was assayed in sheep RBC (sRBC) and chicken RBC (cRBC). The absorbance values measured at 570 nm from the supernatant of lysed RBC in the presence of PBS and 0.1% Triton X-100 were determined as 0 and 100% hemolysis, respectively. Results were expressed as means ± SD (*n* = 4).

The hemolytic activity of mastoparan-AF was measured. Figure 3 shows mastoparan-AF with little hemolytic activity on sheep RBCs even at the highest concentration tested (256 μg mL^−1^). At lower concentrations (32 μg mL^−1^ or below), mastoparan-AF caused little or mild hemolysis on chicken RBCs. However, at higher concentrations (64 μg mL^−1^ or above), mastoparan-AF exhibited some hemolytic activity on chicken RBCs in a dose-dependent manner.

### 3.4. Antibacterial Activities of Mastoparan-AF

We examined the antibacterial effect against the hemolytic *E. coli* O157:H7 (ATCC 43894), and two clinical isolates (232 and 237) of hemolytic *E. coli* from dogs with severe diarrhea, along with a recombinant *E. coli* JM109 carrying an ampicillin-resistant (*Amp^R^*) plasmid pAcUW21 (JM109/pAcUW21). As a comparison, antibacterial activity on *Staphylococcus aureus* subsp. *aureus* that had been examined in our previous study [6] was included and analyzed in parallel. The antibacterial activity assay of mastoparan-AF was performed according to a colorimetric method used for mastoparan-AF and other antimicrobial peptides [6,28,29]. Mastoparan-AF showed antibacterial activity against these Gram-positive and Gram-negative bacteria. Table 1 lists the results. Among five bacteria tested (including one non-pathogenic *Amp^R^* andfour pathogenic bacteria), Gram-negative *E. coli* 237 (median MIC and MBC at 4 μg mL^−1^) and 232 (median MIC and MBC at 8 μg mL^−1^) isolates were the most sensitive to mastoparan-AF. The other bacteria tested were inhibited and killed by mastoparan-AF at median MICs and MBCs ranged from 16 to 32 μg mL^−1^ (Table 1). In particular, hemolytic *E. coli* O157:H7 and *S. aureus* subsp. *aureus* were inhibited by mastoparan-AF with a median MIC at 16 μg mL^−1^ and 32 μg mL^−1^, respectively, and killed by it with an MBC at 32 μg mL^−1^ (Table 1).

### 3.5. Antibiotics Susceptibility of Bacteria

Hemolytic *E. coli* O157:H7 (ATCC 43894) and the aforementioned four other bacteria (*Staph. aureus* subsp. *aureus*, two clinical isolates of *E. coli*, and *E. coli* JM109/pAcUW21) were tested for their antibiotic susceptibility or resistance according to the CLSI guidelines. The MIC of each antibiotic is defined as the lowest concentration that completely inhibited bacterial growth, which is of the same definition as the MIC of mastoparan-AF described above. As shown in Table 2, *E. coli* O157:H7 and JM109/pAcUW21 strains are highly resistant to penicillins tested in this study, with their MIC > 1024 for both ampicillin (AMP) and ticarcillin (TIC), MIC > 1024/2 for TIC/clavulanic acid (CLA), and respective MIC of 64/32 (*E. coli* O157:H7) and 32/16 (JM109/pAcUW21 strain) for amoxicillin (AMX)/CLA. Moreover, *E. coli* O157:H7 is resistant to most antibiotics tested here, with MIC of 16 for doxycycline (DOX) (a tetracycline), >32 for trimethoprim (TMP)/sulfamethoxazole (SXT) (sulfonamides), >1024 for chloramphenicol (CHL), 8 for cefazolin (CFZ) (a cephalosporin), and with intermediate sensitivity to another cephalosporin, cefoxitin (FOX) (MIC of 16). Two *E. coli* clinical isolates, 232 and 237, from dogs, however, showed variable resistance to different antibiotics. Remarkably, *E. coli* isolate 232 is highly resistant to CFZ and FOX (cephalosporins), with respective MIC of 256 and 128, whereas isolate 237 is highly resistant to DOX (a tetracycline) (MIC = 64) and TMP/SXT (sulfonamides) (MIC > 32). In comparison, *S. aureus* subsp. *aureus* is categorized as resistant to penicillin antibiotics tested here, with MIC values of 256 for AMP, 32 for TIC, 8/4 for AMX/CLA, and 256/2 for TIC/CLA, and to DOX (a tetracycline) with MIC of 16. Unlike *E. coli* O157:H7, *S. aureus* subsp. *aureus* is sensitive to TMP/SXT and CHL. All five bacteria examined here are sensitive to tested aminoglycosides: amikacin (AMK), and gentamicin (GEN).

### 3.6. The Effect of Mastoparan-AF on the Morphology of Hemolytic E. coli O157:H7

**Figure 4:**
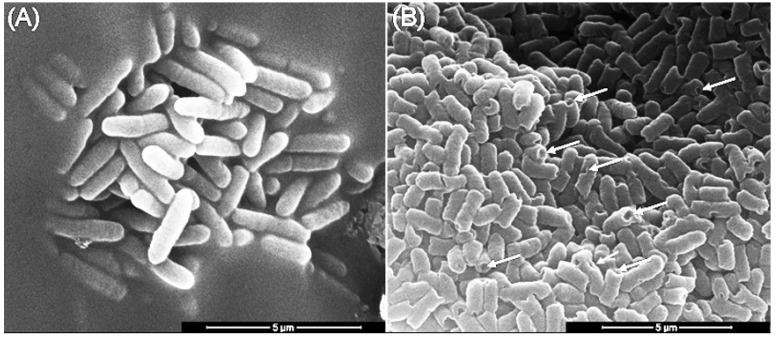
The effects of mastoparan-AF on hemolytic *E. coli* O157:H7 investigated by SEM. (**A**) Untreated hemolytic *E. coli* O157:H7. (**B**) Hemolytic *E. coli* O157:H7 were treated with mastoparan-AF at 2 × MIC (32 μg mL^−1^) for 1 h. Abnormal dents and large perforations (full opening) at apical ends (indicated by arrows) appeared on the surface of bacteria.

**Figure 5:**
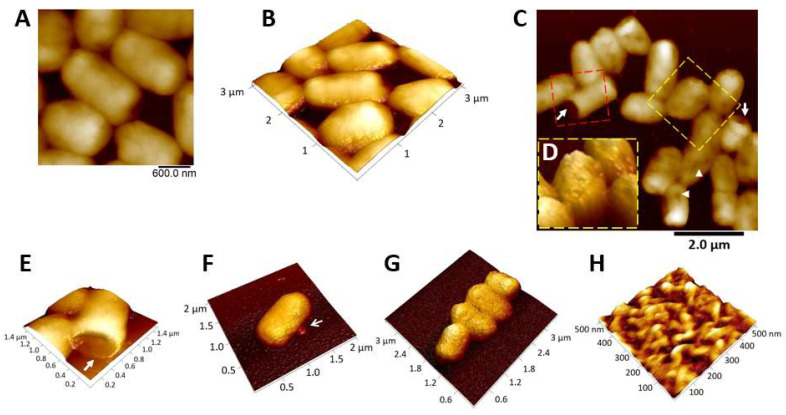
The topology of mastoparan-AF treated-hemolytic *E. coli* O157:H7 analyzed by AFM. (**A**) Two-dimensional (2D) and (**B**) three-dimensional (3D) images show smooth cell surfaces of untreated hemolytic *E. coli* O157:H7. (**C**) A 2D image of mastoparan-AF (32 μg mL^−1^)-treated hemolytic *E. coli* O157:H7. Abnormal perforations and dents on the surface of bacteria are indicated by arrows and arrowheads, respectively. The 3D images focusing on two highlighted areas of (**C**), respectively, reveal (**D**) a rough cell surface and (**E**) a hollow tube resulting from perforations at apical ends. (**F**) A 3D image shows a mastoparan-AF-treated bacterium with a budding vesicle. (**G**) A 3D image shows mastoparan-AF-treated bacteria with a wrinkled or rough surface. (**H**) Magnification of portion of (**G**) displays, in high resolution, the surface roughness of a mastoparan-AF-treated bacterium.

**Figure 6:**
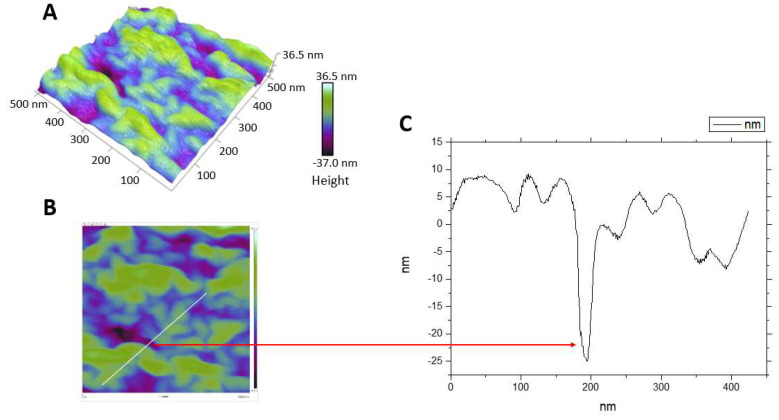
Membrane corrugation and invagination on the cell surface of mastoparan-AF-treated hemolytic *E. coli* O157:H7 investigated by AFM. (**A**) Mastoparan-AF-treated hemolytic *E. coli* O157:H7 with rough surface was viewed from the top. The height of cell surface ranges from 36.5 nm to −37 nm. Purple and dark purple colors indicate deep pits or invaginated areas. (**B**) Cross-sectional analysis was performed (marked in white). (**C**) A cross-section view reveals membrane corrugation and invagination. An invaginated pit was measured as 25 nm deep.

The effects of mastoparan-AF on the morphology of hemolytic *E. coli* O157:H7 were investigated by SEM. Untreated bacteria were rod-shaped with a smooth surface (Figure 4A). After mastoparan-AF treatment at 2 × MIC (32 μg mL^−1^) for 1 h, irregular dents, and full perforations at apical ends appeared on the surface of *E. coli* O157:H7 (Figure 4B). Mastoparan-AF-treated (32 μg mL^−1^) hemolytic *E. coli* O157:H7 was further analyzed by AFM. In contrast to the smooth surface of untreated bacteria (Figure 5A,B), dents (Figure 5C), large perforations at apical ends (Figure 5C,E), vesicle budding (Figure 5F), and rough (or wrinkled) surface (Figure 5D,G) were observed in mastoparan-AF-treated bacteria. Large perforations that preferentially occurred at apical ends may turn bacteria into hollow tubes (Figure 5E). A high-resolution image of the rough surface of mastoparan-AF-treated bacteria reveals membrane corrugation and invagination (Figure 5H). Invaginated areas are irregularly shaped, ranging from 25 to 150 nm in length, 25 to 50 nm in width, and 2.5 to 37 nm in depth (Figure 6A–C). Cross-sectional analysis shows membrane corrugation and invagination, as well as pits or pores, resulting from deep invagination. One of such invaginated pits was measured as 25 nm deep (Figure 6C).

### 3.7. Physicochemical Properties of Mastoparan-AF and Other Mastoparans

**Figure 7:**
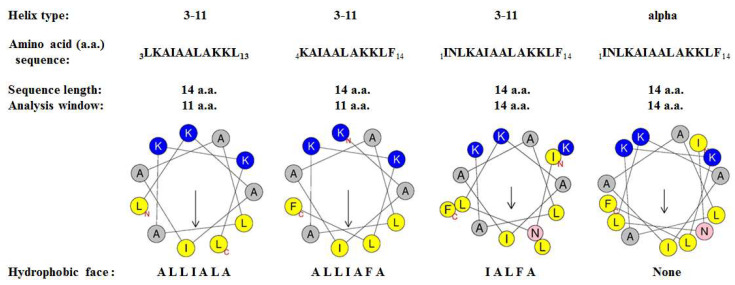
Helical wheel plots of mastoparan-AF. Helical wheel plots were drawn by using the Heliquest software available online [https://heliquest.ipmc.cnrs.fr/], accessed on 4 July, 9 July and 26 July 2021. N denotes the amino terminus and C represents the carboxyl terminus. Amino acid residues are displayed in one letter code. The directions of the hydrophobic moment are indicated by arrows and the magnitudes are proportional to their lengths. Charged, uncharged polar, weak hydrophobic, and strong hydrophobic amino acid residues are shown in blue, pink, grey, and yellow, respectively. Representative 3–11 helical structures with different hydrophobic face versus one α helical structure without hydrophobic face are shown.

Considering that helical structure is required for the activities of mastoparans [17,19], we employed an online software, Heliquest (https://heliquest.ipmc.cnrs.fr/), on 4 July, 9 July and 26 July 2021 [33], to assess the physicochemical properties of mastoparan-AF under different helical structures and compared these parameters with those of other mastoparans. A hydrophobic face consists of at least five adjacent hydrophobic amino acid residues presented on a helical wheel. With a net positive charge of three, mastoparan-AF may adopt several forms of 3–11 helix, including three forms with hydrophobic faces as IALFA, ALLIALA, and ALLIAFA, respectively. Alternatively, it may possibly adopt an α-helix without any hydrophobic face (Figure 7, Table 3). As shown in the helical wheel plots, these 3–11 helices of mastoparan-AF are amphipathic helices comprising 11 amino acid residues evenly distributed in three helical turns, which are slightly different from the α-helix structure (Figure 7). Similarly, other mastoparan homologs, including mastoparan-A, -B, -D, -M, and -V, could also form several 3–11 helix structures, with three of them each containing a hydrophobic face, whereas they each could potentially form one α-helix structure without any hydrophobic face (Table 3). Among all mastoparans analyzed here, their three forms of amphipathic 3–11 helix structures (each forming a hydrophobic face) share higher hydrophobic moments than their α-helix counterparts (Table 3). In addition, according to the data output generated by using Heliquest software, helical wheel plots of 3–10 helix and π-helix types among all mastoparans do not display any continuous hydrophobic face.

## 4. Discussion

Here we reported the cloning of the full-length cDNA encoding the precursor polypeptide of mastoparan-AF (Figure 1). Mastoparan-AF exhibits antibacterial activity against multiple antibiotic-resistant Gram-negative *E. coli* O157:H7, two other *E. coli* clinical isolates, and Gram-positive *S. aureus* subsp. *aureus*, with little or limited hemolytic activity on sheep and chicken erythrocytes (Table 1 and Table 2, and Figure 3). Mastoparan-AF treatment correlates proportionally with membrane permeabilization in hemolytic *E. coli* O157:H7 (Figure 2). Moreover, examining by SEM and AFM, our results illustrate that mastoparan-AF treatment may lead to multiple membrane disruption patterns (Figure 4, Figure 5 and Figure 6). Physicochemical analysis indicates that mastoparans may favorably adopt 3–11 helices to facilitate membrane interaction (Figure 7).

The anionic prosequence of prepromastoparan-AF, as presented in Figure 1 of this study, is rich in alanine (A), aspartate (D), glutamate (E), and proline (P), as found in the prosequence of prepromastoparan B [27]. The positively charged lysine (K) residue is located at position 4, 11, and 12 in the mature mastoparan-AF (Figure 1), which is the same as in mastoparan-M [10]. The 11-bp “CATCATGAAGA” sequence (−4 to +7) flanking the translation initiation codon, which is quite different from that of other genes from *Vespa* spp., is very conserved among prepromastoparan homologs of *Vespa* spp. (Figure 1) [27,34]. Within −10 to +4, prepromastoparan-AF and prepromastoparan-B mRNAs share the identical sequence 5′-AAAAACCATCATGA, which is more similar to *Drosophila* consensus than to vertebrate consensus [30,31] sequences. Moreover, CANCAUG (−4 to +3) appears to be the common Kozak-like consensus sequence among prepromastoparan, *Drosophila,* and vertebrate mRNAs. A previous study has shown that “RYMRMVAUGGC“ (−6 to +5) (R = A or G; Y = U or C, M = A or C, and V = A, C, or G) can facilitate start codon recognition and increase translation efficiency of mammalian mRNAs [35]. Taken altogether, prepromastoparan mRNAs fulfill most features of highly efficient translation initiation sites except for the −2 position (T in prepromastoparan and A or C in *Drosophila* and mammalian consensus), and the +4 position (A in prepromastoparan and *Drosophila* consensus; G in mammalian consensus). Notably, prepromastoparan-AF (this study) and prepromastoparan-B mRNA [6,27] sequences that are identical from −13 to +7 contain a repetitive poly(A) stretch between −13 to −6, which might function as a 5′-poly(A) leader at the 5′-untranslated region, critical for bypassing the cap-dependent translation and in turn having an advantage in cap-independent translation initiation [36]. Our attempt to express recombinant mastoparan-AF in the cost-effective prokaryotic (bacterial) system was not fruitful. Mastoparan-AF, being an antibacterial peptide, may be too toxic for bacteria. Unfrequent codon usages of cloned sequence for the bacteria could also account for its lack of expression. In the future, it may be feasible to express recombinant mastoparan-AF in an insect expression system since mastoparan-AF is an insect peptide. The mammalian expression system is another option to consider based on the lower toxicity observed in sheep and human RBCs. Alternatively, we may try to express prepromastoparan-AF and produce mastoparan-AF by inserting a convenient cleavage site between prosequence and mature mastoparan-AF. However, the −2 and +4 variations from insect and mammalian consensus translation initiation sites may be tested and codons may be optimized for the respective system.

Previously, we examined synthetic mastoparan-AF for its membrane permeabilization effect on *E. coli* BL21 (non-pathogen) and hemolytic activity in a higher concentration range [6]. In this study, we investigated its membrane permeabilization effect on the pathogenic *E. coli* O157:H7 and focused on the hemolytic activity characterizations in more detail within the actual antibacterial concentration range of mastoparan-AF. In comparison, mastoparan-AF exerts around 40% lower membrane permeabilization activities on *E. coli* O157:H7 (Figure 2) than BL21 [6] at 6.4 and 12.8 μg/mL. In our membrane permeabilization assay, the time frames for antimicrobial peptide preincubation and substrate conversion were optimized according to our previous study [6], which may vary with different antimicrobial peptides [37,38]. The hemolytic activities of mastoparan-AF presented in this study for chicken and sheep RBCs are consistently comparable with those of our previous study [6]. The hemolytic activities of mastoparan-AF among sheep appear much lower than chickens and deserve further investigation on various commercial breeds for veterinary applications. It is worth noting that hemolytic activities of a variety of antimicrobial agents in many studies may be based on RBCs of different species, such as human RBCs [6,28] and sheep RBCs [6,39], and the range of variations within the same or between species should be taken into consideration when making comparisons. For example, a membrane-disrupting antibacterial agent, selected from a group of cationic hyperbranched synthetic polymers called molecular umbrellas, has shown a low hemolytic activity on sheep RBCs, with 50% hemolysis at a concentration (HC_50_) around 5000 μg/mL and high selectivity indexes (HC_50_/MIC) of 640 and 1280, respectively, for a multiple drug-resistant *E. coli* strain (MIC 7.8 μg/mL) and a drug-sensitive *S. aureus* (MIC 3.9 μg/mL) [39]. In our study, mastoparan-AF exerts only 2% hemolysis on sheep RBCs at 256 μg/mL (Figure 3). We expect that mastoparan-AF would have high HC_50_ on sheep RBCs and potentially high selectivity indexes for *E. coli* clinical isolates in this study. The actual comparisons between mastoparan-AF and other antibacterial agents, however, require extensive investigations on the range of selectivity indexes based on the same animal species. The outer leaflet of the RBC membrane mainly comprises electrically neutral zwitterionic phospholipids, *i.e.*, phosphatidylcholine or sphinogomyelin, and cholesterol, and membranes with this feature are more resistant to cationic antimicrobial peptides [24]. In contrast, cationic antimicrobial peptides preferentially bind to negatively charged bacterial membranes through electrostatic interactions [24]. This can explain the limited hemolytic activity of mastoparan-AF within 64 μg mL^−1^ on sheep and chicken RBCs as shown in Figure 3, and on human RBCs in our previous study [6], at which concentration all bacteria tested in this study were sensitive to mastoparan-AF, a lysine-rich cationic peptide. Similarly, the variable hemolytic activity of mastoparan-AF on chicken (Figure 3) and human [6] RBCs at higher concentrations, as well as the resistance of sheep RBCs (Figure 3) to mastoparan-AF treatment, could be due to the different membrane compositions of RBCs in these three species. In fact, the total percentage of electrically neutral sphinogomyelin and phosphatidylcholine in the lipid contents of sheep RBC membrane is higher than that of human RBC [40,41], which may explain the resistance of sheep RBCs to mastoparan-AF. We suggest that the range of hemolytic activity should be evaluated in different populations, which could have variable membrane compositions of RBCs, if mastoparan-AF is considered for future clinical applications in humans.

Based on our data (Table 2), the hemolytic *E. coli* O157:H7 is much more resistant to penicillins (MIC > 1024 μg mL^−1^), sulfonamides (MIC > 32 μg mL^−1^), and chloramphenicol (MIC > 1024 μg mL^−1^) than the Gram-positive pathogen *S. aureus* subsp. *aureus*. In contrast, mastoparan-AF exerts lower MIC and MBC of 16 and 32 μg mL^−1,^ respectively, on this hemolytic pathogen (Table 1). At such concentrations, the membrane permeabilization is over 80% of the positive control (Figure 2). In addition, *E. coli* clinical isolates, including 232 that is highly resistant to cephalosporins and AMX/CLA (penicillins), and 237 to AMP and TIC (penicillins), DOX (a tetracycline) and sulfonamides, turn out to be very sensitive to mastoparan-AF, with the average MIC/MBC of 8/8 and 4/4, respectively. Consistent with our previous study, mastoparan-AF, with the MIC/MBC ranging from 16 to 32 μg mL^−1^ for *S. aureus* subsp. *aureus* appeared to have comparable or better antibacterial activity than mastoparans from other wasp species [6]. *E. coli* O157:H7 is marginally more sensitive to mastoparan-AF inhibition than *S. aureus* subsp. *aureus* (Table 1). Mastoparan-AF treatment, as examined by SEM and AFM (Figure 4, Figure 5 and Figure 6), could damage the cell surface of hemolytic *E. coli* O157:H7, rendering membrane permeabilization (Figure 2) and cell death (Table 1).

In this study, detailed AFM images unveil multiple membrane disruption patterns on mastoparan-treated *E. coli* O157:H7. Earlier on, some studies have used AFM to examine antimicrobial peptides-treated non-pathogenic *E. coli* [42,43,44,45]. Among these studies, melittin (from honeybee)-treated *E. coli* HB101 strain (non-pathogen), which appeared with “grooves” and “pore-like lesions” at the apical ends, “surface roughness or corrugation”, and a “blebbing-like protrusion” at one apical end [42], may share partly similar features with mastoparan-AF-treated hemolytic *E. coli* O157:H7 presented in this study (i.e., dents, pits, surface corrugation, and vesicle budding). In addition, our cross-sectional analysis further unveils the rough surface of mastoparan-AF-treated hemolytic *E. coli* O157:H7 as the coexistence of membrane corrugation and invagination, resulting in pits or pores (25 nm in depth or deeper) with irregular shapes or sizes (Figure 6). The surface roughness and irregular pits or pores formation may fit carpet model poration that may lead to membrane corrugation and disruption (invaginated pores) or toroidal model poration that may form irregular pores [26]. Previous studies on other mastoparans-treated artificial giant vesicles and simulated vesicles interacting with melittin have indicated vesicle budding is facilitated under higher peptide concentrations [23,46]. Based on a simulation study, binding and penetration of peptide monomers may induce positive membrane curvature and vesicle budding, whereas peptide oligomers may induce negative membrane curvature and membrane invagination [23]. Remarkably, the hollow tube resulting from completely perforated apical ends (Figure 4 and Figure 5C,E) shown in our study indicates that both the outer membrane and inner membrane at apical ends of *E. coli* O157:H7 are damaged by mastoparan-AF, and this drastic disruption pattern appears distinct from and more serious than that caused by melittin [42]. However, in both cases, the apical ends of *E. coli* are prone to cationic peptide damage. This could be explained by the cardiolipin domain distribution at the inner membrane of *E. coli* apical ends [47], and the negatively charged phospholipid may attract cationic antimicrobial peptides such as mastoparan-AF (this study) or melittin [42] to exert disruption.

Physicochemical properties of mastoparan-AF and five other mastoparan homologs obtained in this study by using Heliquest online software have, respectively, revealed three forms of 3–11 helix, each exhibiting an uninterrupted hydrophobic face (comprising at least five adjacent hydrophobic amino acid residues) with a higher hydrophobic moment than the α-helix counterpart, which has no hydrophobic face (Figure 7 and Table 3). However, alternative interpretations are possible. For example, after the N residue, a potential hydrophobic face may form as LIALFA (from N-terminal to C-terminal) for an α-helix wheel (Figure 7) if these six residues space evenly. On the other hand, it is known that lysine residues flanking the transmembrane segments of membrane proteins may “snorkel” the positively charged amino group toward a more polar region and bury the aliphatic chain in the membrane [48]. If we think of mastoparans as short flexible peptides with potentially helical and rotational dynamics on the membrane, “snorkeling” of lysine residues in mastoparans could form an alternative hydrophobic face on the side of their aliphatic chains for 3–11, α, 3–10, or π helices. It is known that a helical structure is required for the activities of mastoparans [17,19]. A previous study has indicated that a synthetic amphipathic peptide isomer with the highest hydrophobic moment than the other isomers presents with the best membrane interaction ability and forms stable membrane pores with the strongest membrane damage [49]. Therefore, based on our physicochemical analysis results, it is feasible that mastoparan-AF, -A, -B, -D, -M, and -V may adopt dynamically favorable 3–11 helix structures (instead of α-helix) to facilitate membrane interaction, and thereby result in membrane disruption on the surface of bacteria.

## 5. Conclusions

In conclusion, prepromastoparan-AF and other prepromastoparan mRNAs share an 11-bp conserved sequence (CATCATGAAGA, −4 to +7) flanking the initiation codon, and exhibit a 5′-poly(A) leader sequence at the 5′-UTR known for the advantage in cap-independent translation. Mastoparan-AF kills multi-antibiotic resistant hemolytic *Escherichia coli* O157:H7 through multiple membrane disruption patterns, including large perforations (full opening) at apical ends (hollow tubes), vesicle budding, forming dents, and membrane corrugation and invagination leading to irregular pits or pores. Our physicochemical property analysis data indicate that mastoparans may favorably adopt 3–11 helices to facilitate membrane interaction, and thereby result in membrane disruption on the surface of bacteria. This is the first report about the physicochemical adaptation of 3–11 amphipathic helices among mastoparans or antimicrobial peptides. Considering that *E. coli* O157:H7 and clinical isolates are highly resistant to multiple classes of antibiotics, mastoparan-AF, with little or mild effect on animal RBCs, could be an effective and alternative treatment to combat hemolytic *E. coli* O157:H7 and other pathogenic *E. coli*.

## Figures and Tables

**Table 1:** Antibacterial activity of mastoparan-AF against *Escherichia coli* O157:H7, *E. coli* clinical isolates, and *Staphylococcus aureus* subsp. *aureus*.

Microorganisms	Mastoparan-AF (μg mL^−1^)			
	MIC ^a^ (μg mL^−1^)		MBC ^b^ (μg mL^−1^)	
	Range	M ^c^	Range	M
Gram-positive bacteria				
*S. aureus* subsp. *aureus*	16–32	32	32	32
Gram-negative bacteria				
*E. coli* JM109 pAcUW21 (*Amp^R^*) ^d^	16	16	16	16
*E. coli* O157:H7	16–32	16	16–32	32
*E. coli* 232 ^e^	4–8	8	4–8	8
*E. coli* 237 ^e^	4	4	4	4

^a^ minimum inhibitory concentration. ^b^ minimum bactericidal concentration. ^c^ Medians from three independent experiments performed in duplicates. ^d^ Non-pathogenic strain carrying an ampicillin-resistant gene (*Amp^R^*) in its plasmid. ^e^ Clinical isolates from diarrhetic dogs.

**Table 2:** Minimum inhibitory concentration ^a^ of antibiotics against *Escherichia coli* O157:H7, two clinical isolates, and *Staphylococcus aureus* subsp. *aureus*.

	Penicillins				Cephalosporins		Aminoglycosides		TET	Sulfa	CHL
Bacteria	AMP ^b^	AMX/ CLA	TIC	TIC/ CLA	CFZ	FOX	AMK	GEN	DOX	TMP/ SXT	CHL
*E. coli* strain											
JM109 pAcUW21 (*Amp*R)	>1024 **^R^**	32/16 **^R^**	>1024 **^R^**	>1024/2 **^R^**	32 **^R^**	16 ^I^	4 ^S^	1 ^S^	2 ^S^	16 **^R^**	8 ^S^
O157:H7	>1024 **^R^**	64/32 **^R^**	>1024 **^R^**	>1024/2 **^R^**	8 **^R^**	16 ^I^	4 ^S^	1 ^S^	16 **^R^**	>32 **^R^**	>1024 **^R^**
232	16 ^I^	128/64 **^R^**	8 ^S^	32/2 ^I^	256 **^R^**	128 **^R^**	4 ^S^	1 ^S^	8 ^I^	8 **^R^**	8 ^S^
237	>1024 **^R^**	16/8 ^I^	>1024 **^R^**	64/2 ^I^	4 ^I^	8 ^S^	4 ^S^	1 ^S^	64 **^R^**	>32 **^R^**	8 ^S^
*S. aureus* subsp. *aureus*	256 **^R^**	8/4 **^R^**	32 **^R^**	256/2 **^R^**	4 ^N^	8 ^N^	4 ^S^	2 ^S^	16 **^R^**	0.25 ^S^	8 ^S^

^a^ concentration unit: μg mL^−1^. ^b^ ampicillin (AMP); amoxicillin/clavulanic acid (AMX/CLA); ticarcillin (TIC); ticarcillin/ clavulanic acid (TIC/CLA); cefazolin (CFZ); cefoxitin (FOX); amikacin (AMK); gentamicin (GEN); tetracycline (TET); doxycycline (DOX); sulfonamide (Sulfa); trimethoprim/sulfamethoxazole (TMP/SXT); chloramphenicol (CHL). Bacteria were categorized as **^R^** resistant, ^I^ intermediate or ^S^ sensitive to an antibiotic based on CLSI M100 performance standards for antimicrobial susceptibility testing. ^N^ No data from CLSI.

**Table 3:** Helical types and physicochemical properties of mastoparans.

Peptide Name	Helical Type	Sequence	Analysis Window	Hydrophobicity H	Hydrophobic Moment μH	Net Charge z (+)	Hydrophobic Face
Mastoparan-AF	3–11	_1_INLKAIAALAKKLF_14_	14 aa	0.583	0.418	3	IALFA
	3–11	_3_LKAIAALAKKL_13_	11 aa	0.470	0.623	3	ALLIALA
	3–11	_4_KAIAALAKKLF_14_	11 aa	0.478	0.625	3	ALLIAFA
	α	_1_INLKAIAALAKKLF_14_	14 aa	0.583	0.400	3	none
Mastoparan-A	3–11	_1_IKWKAILDAVKKVI_14_	14 aa	0.549	0.532	3	IVWIL
	3–11	_3_WKAILDAVKKV_13_	11 aa	0.461	0.747	2	AAVIVML
	3–11	_4_KAILDAVKKVI_14_	11 aa	0.420	0.718	2	AAVIVIL
	α	_1_IKWKAILDAVKKVI_14_	14 aa	0.549	0.544	3	none
Mastoparan-B	3–11	_1_LKLKSIVSWAKKVL_14_	14 aa	0.561	0.404	4	IALLV
	3–11	_3_LKSIVSWAKKV_13_	11 aa	0.495	0.622	3	WVIALV
	3–11	_4_KSIVSWAKKVL_14_	11 aa	0.495	0.622	3	WVIALV
	α	_1_LKLKSIVSWAKKVL_14_	14 aa	0.561	0.404	4	none
Mastoparan-D	3–11	_1_INLKAIAAFAKKLL_14_	14 aa	0.583	0.419	3	IALLA
	3–11	_3_LKAIAAFAKKL_13_	11 aa	0.478	0.628	3	AFLIALA
	3–11	_4_KAIAAFAKKLL_14_	11 aa	0.478	0.628	3	AFLIALA
	α	_1_INLKAIAAFAKKLL_14_	14 aa	0.583	0.402	3	none
Mastoparan-M	3–11	_1_INLKAIAALAKKLL_14_	14 aa	0.576	0.416	3	IALLA
	3–11	_3_LKAIAALAKKL_13_	11 aa	0.470	0.623	3	ALLIALA
	3–11	_4_KAIAALAKKLL_14_	11 aa	0.470	0.623	3	ALLIALA
	α	_1_INLKAIAALAKKLL_14_	14 aa	0.576	0.399	3	none
Mastoparan-V	3–11	_1_INWKGIAAMAKKLL_14_	14 aa	0.560	0.421	3	IAWLA
	3–11	_3_WKGIAAMAKKL_13_	11 aa	0.449	0.622	3	MLIAWA
	3–11	_4_KGIAAMAKKLL_14_	11 aa	0.399	0.602	3	MLIALA
	α	_1_INWKGIAAMAKKLL_14_	14 aa	0.560	0.419	3	none

## Data Availability

The data supporting our reported findings are presented in the manuscript.
